# Reference Ranges for Trabecular Bone Score in Australian Men and Women: A Cross‐Sectional Study

**DOI:** 10.1002/jbm4.10133

**Published:** 2019-01-15

**Authors:** Kara B Anderson, Kara L Holloway‐Kew, Didier Hans, Mark A Kotowicz, Natalie K Hyde, Julie A Pasco

**Affiliations:** ^1^ Deakin University, School of Medicine Geelong Australia; ^2^ Center of Bone Diseases, Bone & Joint Department Lausanne University Hospital Lausanne Switzerland; ^3^ Barwon Health Geelong Australia; ^4^ Department of Medicine Western Campus The University of Melbourne St Albans Australia; ^5^ Department of Epidemiology and Preventive Medicine Monash University Melbourne Australia

**Keywords:** EPIDEMIOLOGY, FRACTURE RISK ASSESSMENT, DXA, OSTEOPOROSIS, SCREENING

## Abstract

Trabecular bone score (TBS) is a novel method for indirectly assessing trabecular microarchitecture at the lumbar spine, providing information complementary to areal BMD. However, limited reference ranges exist for the normative distribution of TBS, particularly in men. The aim of this study was to develop such a reference range in Australian men and women. This study included 894 men and 682 women (aged 24 to 98 years) enrolled in the Geelong Osteoporosis Study. TBS was determined retrospectively by analysis of lumbar spine DXA scans (Lunar Prodigy) using TBS iNsight software (version 2.2). Multivariable regression techniques were used to determine best‐fit models for TBS incorporating age, height, and weight. Age‐related differences in TBS were best modelled with a linear relationship in men and a cubic relationship in women. Combined best‐fit models for TBS included age and weight in men, and age and height in women. This study provides normative reference ranges for TBS in Australian men and women, and further indicates that TBS may identify individuals at risk for fracture despite normal BMD. © 2018 The Authors. *JBMR Plus* Published by Wiley Periodicals, Inc. on behalf of the American Society for Bone and Mineral Research.

## Introduction

Osteoporosis is a condition defined by low bone mass and the deterioration of bone microarchitecture resulting in increased bone fragility and a subsequent increase in fracture risk.^1^, ^2^ The current gold‐standard for the diagnosis of osteoporosis and determination of fracture risk is bone mineral density (BMD), measured using dual X‐ray absorptiometry (DXA) at the hip or lumbar spine. However, evidence has emerged to suggest that although individuals considered to have osteoporosis by BMD are at high risk for fracture, the population burden of fracture actually lies in those without osteoporosis.^3^, ^4^ There are other factors that contribute to fracture risk which are not captured by BMD. One such parameter is trabecular microarchitecture, including trabecular distribution and connectivity, which can be indirectly measured by trabecular bone score (TBS).^5^ The TBS software analyses gray‐level pixel distribution in the lumbar spine DXA image used to calculate BMD, and assesses the subsequent variation to produce a unitless score that reflects the quality of trabecular microarchitecture.^6^ TBS has been associated with vertebral and other osteoporotic fractures in cross‐sectional studies, and predicts fracture in prospective studies.^7^, ^8^ However, few studies have published normative data in the form of an age‐related reference range for TBS, particularly in men, and these studies have primarily utilized clinical populations and volunteers,^9^, ^10^ which limit their generalizability to the underlying population.^11^ The aim of the current study was to develop age‐related normative reference ranges for TBS in both men and women using an Australian, randomly‐selected cohort.

## Subjects and Methods

### Study region

This study utilized cross‐sectional data from a cohort of men and women assessed as part of the Geelong Osteoporosis Study (GOS),^12^ which included residents of the Barwon Statistical Division in southeastern Australia. Baseline recruitment of women began in 1993 (*n* = 1494), with recruitment of a subsequent group of men from 2001 (*n* = 1540). Participants were randomly selected in age‐stratified samples from the Australian electoral rolls to ensure at least 100 men and women in each 5‐year age‐group from 20 to 69 years, and 200 for ages 70 to 79 years and 80+ years. Electoral roll registration is compulsory in Australia, resulting in a near‐comprehensive sampling frame of adults aged over 18 years. Participation at baseline for the women and men were 77% and 67%, respectively. At the 10‐year follow‐up, the cohort was supplemented with a further 246 women aged 20 to 29 years on the 2005 electoral roll, in order to continue with a cohort spanning the complete age range. The full protocol for the GOS has been published elsewhere.^12^

### Participants

Data for the current study were drawn from the 5‐year follow up visit for men (2006–2011) and the 15‐year follow up visit for women (2011–2014). Of the 1540 men recruited at baseline, 141 had died before the 5‐year follow‐up, 41 had left the region, 16 were unable to provide informed consent, 139 were not able to be contacted, and 225 declined, resulting in 81% of eligible men participating in the follow‐up. Similarly, of the 1750 women recruited at baseline and 10‐year follow‐up, 397 had died prior to the 15‐year follow‐up, 94 had left the study region, 96 were unable to participate due to advancing age, illness, or language barriers, 125 were not able to be contacted, and 177 declined, resulting in 48% of eligible women participating in the follow‐up. Of 978 men and 849 women eligible for inclusion in the study, 68 men and 119 women did not have a lumbar spine DXA scan for TBS determination, and 16 men and 48 women were outside the BMI range of TBS (15 to 37 kg/m^2^), leaving 894 men and 682 women available for analysis. All participants provided written, informed consent. The study was approved by the Barwon Health Human Research Ethics Committee.

### Participant measures

Lumbar spine BMD (L_2_–L_4_) was determined using DXA (Lunar Prodigy Pro; GE Healthcare, Madison, WI, USA). TBS (L_1_–L_4_) was retrospectively calculated from the same DXA scans using TBS iNsight software (version 2.2; Medimaps Group, Geneva, Switzerland). The coefficient of variation (%) for TBS was 1.93%. Height was measured without shoes using a wall‐mounted stadiometer to the nearest 0.1 cm; weight was measured in a hospital gown or minimal clothing on electronic scales to the nearest 0.1 kg. Body mass index (BMI) was calculated as weight (kg) divided by height (m) squared. Previous low‐trauma fracture (defined as any fracture from standing height or less, other than those of the skull, fingers, and toes) and use of antiresorptive therapy including bisphosphonates and denosumab were determined by self‐report, with fractures being confirmed radiologically where possible.

### Statistical analysis

For both men and women, TBS values were normally distributed and thus means and standard deviations (SDs) were calculated for each age decade from 20 to 79 years, and for the group aged 80 years and over. Young adult mean and standard deviation (20 to 39 years) were determined to allow calculation of TBS *T*‐scores. Fitted line plots were produced to represent the distribution of TBS by age, weight, and height separately, and best subsets regressions were used to develop multivariable models for TBS in association with age, height, and weight. Linear, quadratic, and cubic models were each tested. *T*‐scores were used to group participants based on equivalent BMD *T*‐score cutpoints, with TBS *T*‐scores greater than −1.0 classified as normal microarchitecture, those between −2.5 and −1.0 as partially degraded microarchitecture, and those below −2.5 as degraded microarchitecture. All statistical analyses were performed using MINITAB (version 17; Minitab, Inc., State College, PA, USA).

## Results

### Normative reference data

Table 1 lists the subject characteristics for the cohort of men and women. TBS was inversely associated with age in both men and women, as shown in Table 2. The mean for the young adult group (ages 20 to 39 years) was determined to be 1.333 ± 0.132 for men and 1.399 ± 0.097 in women. These means were used to determine *T*‐scores for TBS for the men and women, respectively. Based upon these calculated *T*‐scores, cutpoints for TBS were determined at −1.0 and −2.5 *T*‐scores to compare with similar thresholds for BMD, and were defined as partially degraded and degraded microarchitecture respectively. In men, these cutpoints were equal to a TBS score less than 1.003 for a determination of degraded microarchitecture, and between 1.003 and 1.201 for a determination of partially degraded microarchitecture. In women, these cutpoints were 1.157 and 1.302, respectively.

**Table 1:** Subject Characteristics

Characteristic	Men (*n* = 894)	Women (*n* = 682)
Age (years), median (IQR)	60.1 (46.4–73.3)	55.3 (42.1–68.1)
Height (cm), mean ± SD	174.8 ± 7.3	162.1 ± 6.5
Weight (kg), median (IQR)	82.0 (74.1–91.8)	70.0 (61.9–81.4)
Lumbar spine BMD (g/cm^2^), mean ± SD	1.294 ± 0.200	1.203 ± 0.184
Trabecular bone score (unitless), mean ± SD	1.226 ± 0.153	1.302 ± 0.149
Prior low trauma fracture, *n* (%)	175 (19.6)	74 (10.8)
Antiresorptive use, *n* (%)	14 (1.6)	19 (2.8)

IQR = interquartile range.

**Table 2:** Trabecular Bone Score in Men and Women per Age Decade

	Men (*n* = 894)		Women (*n* = 682)	
Age (years)	*n*	Trabecular bone score	*n*	Trabecular bone score
20–29	31	1.362 ± 0.113	25	1.406 ± 0.084
30–39	102	1.324 ± 0.137	123	1.398 ± 0.100
40–49	153	1.291 ± 0.113	121	1.381 ± 0.108
50–59	157	1.265 ± 0.124	132	1.293 ± 0.152
60–69	176	1.182 ± 0.152	132	1.244 ± 0.138
70–79	154	1.150 ± 0.142	110	1.208 ± 0.130
80+	121	1.138 ± 0.144	39	1.181 ± 0.152

Values are shown as mean ± SD.

Descriptive characteristics of men and women stratified by TBS category are outlined in Table 3. Based upon the *T*‐score classifications for TBS defined above, 59% (*n* = 531) of men and 54% (*n* = 372) of women had normal microarchitecture; 32% (*n* = 283) of men and 29% (*n* = 197) of women had have partially degraded microarchitecture; and 9% (*n* = 80) of men and 17% (*n* = 113) of women had degraded microarchitecture. Men with degraded or partially degraded microarchitecture were older than men in the normal group. A similar pattern was observed for weight, with those with degraded microarchitecture being heaviest. Mean lumbar spine BMD was lower in the men with degraded microarchitecture (1.212; 95% CI, 1.164 to 1.260), but was not statistically different between the normal or partially degraded groups (1.301; 95% CI, 1.285 to 1.317; and 1.304; 95% CI, 1.279 to 1.329, respectively). In women, those with degraded microarchitecture were older and heavier than the other groups. Lumbar spine BMD was lower and prevalence of prior fracture higher in women with partially degraded and degraded microarchitecture. When grouped by BMD category, 78% (*n* = 698) of men and 73% (*n* = 496) of women had normal BMD, 20% (*n* = 181) of men and 23% (*n* = 155) of women had osteopenia, and 2% (*n* = 15) of men and 5% (*n* = 31) of women had osteoporosis. Height and weight were significantly different between the groups of men, with those with osteoporosis being shorter and lighter, but age was not different; whereas age, height, and weight were all significantly different between the groups for women, with those in the osteoporosis group being older, shorter, and lighter. Similarly, there was no difference in prior fractures between the groups of men, but women with osteoporosis were significantly more likely to have a prior fracture than those in the normal BMD category.

**Table 3:** Descriptive Characteristics, Stratified by TBS T‐Score Category, for Both Men and Women

	Normal microarchitecture	Partially degraded microarchitecture	Degraded microarchitecture	*p*
Men	(*n* = 531)	(*n* = 283)	(*n* = 80)	
Age (years), median (IQR)	53.0 (41.6–66.7)	66.8 (57.1–78.3)	75.0 (65.2–81.9)	<0.001
Height (cm), mean ± SD	175.1 ± 7.3	174.5 ± 7.3	174.2 ± 7.2	0.336
Weight (kg), median (IQR)	79.9 (72.6–89.7)	84.9 (76.5–92.4)	87.4 (81.3–94.1)	<0.001
Lumbar spine aBMD (g/cm^2^), mean ± SD	1.301 ± 0.186	1.304 ± 0.215	1.212 ± 0.219	0.001
TBS (unitless), mean ± SD	1.328 ± 0.088	1.119 ± 0.055	0.933 ± 0.057	<0.001
Any prior low trauma fracture (yes), *n* (%)	99 (18.6)	58 (20.5)	18 (22.5)	0.659
Antiresorptive use (yes), *n* (%)	4 (0.8)	8 (2.8)	2 (2.5)	–
Women	(*n* = 372)	(*n* = 197)	(*n* = 113)	
Age (years), median (IQR)	46.3 (37.1–58.0)	62.6 (52.6–71.4)	69.5 (58.5–76.4)	<0.001
Height (cm), mean ± SD	162.3 ± 6.1	162.5 ± 6.9	161.0 ± 7.1	0.125
Weight (kg), median (IQR)	67.6 (60.6–81.3)	71.7(63.4–80.1)	75.6 (66.1–82.2)	0.004
Lumbar spine aBMD (g/cm^2^), mean ± SD	1.253 ± 0.168	1.159 ± 0.175	1.114 ± 0.196	<0.001
TBS (unitless), mean ± SD	1.437 ± 0.077	1.272 ± 0.049	1.100 ± 0.091	<0.001
Any prior low trauma fracture (yes), *n* (%)	22 (5.9)	33 (16.7)	19 (16.8)	<0.001
Antiresorptive use (yes), *n* (%)	7 (1.8)	3 (1.5)	9 (8.0)	0.001

Values of *p* have been provided for differences between groups.

TBS = trabecular bone score; IQR = interquartile range.

### Multivariate regression modelling

**Figure 1:**
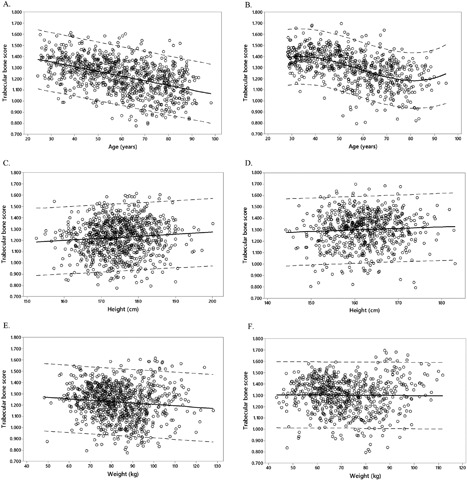
Fitted line plots of trabecular bone score in relation to age (*A*, *B*), height (*C*, *D*), and weight (*E*, *F*). Data for men is presented on the left hand side of the figure (*A*, *C*, *E*) and for women on the right side of the figure (*B*, *D*, *F*). 95% prediction intervals are marked by broken gray lines.

Fitted line plots show the relationships between TBS and age, height, and weight (Fig. 1 *A*–*F*). In men, a linear relationship was observed between TBS and each of these variables, whereas in women, there was a cubic relationship between age and TBS. For men, a negative association was observed between age and TBS (*R* ^2^ = 21.4%, *p* < 0.001), and weight and TBS (*R* ^2^ = 1.0%, *p* = 0.002), whereas a weak positive association was observed between TBS and height (*R* ^2^ = 0.6%, *p* = 0.009). A best‐fit model for TBS included age and weight, In women, there was a negative cubic association between TBS and age (*R* ^2^ = 27.2%, *p* < 0.001). No association was observed between TBS and height (*R* ^2^ = 0.2%, *p* = 0.122), and TBS and weight (*R* ^2^ = 0.0%, *p* = 0.769). The best‐fit model for women included age and height.

### Distribution of TBS within BMD categories

**Figure 2:**
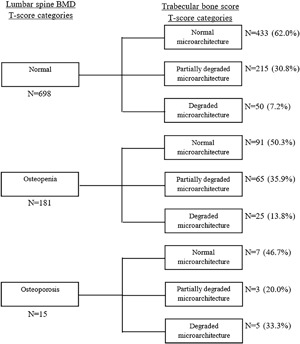
Comparison between lumbar spine BMD *T*‐score categories and trabecular bone score *T*‐score categories in men.

**Figure 3:**
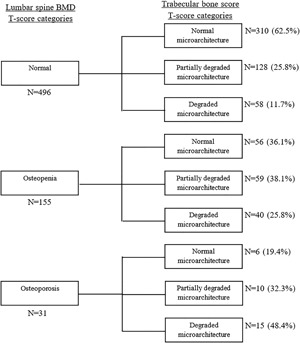
Comparison between lumbar spine BMD *T*‐score categories and trabecular bone score *T*‐score categories in women.

Among 698 men classified as normal on the basis of lumbar spine BMD, 265 (38%) were considered to have partially degraded or degraded microarchitecture on the basis of TBS (Fig. 2). Similarly, of 181 men considered osteopenic by lumbar spine BMD, 25 (14%) were considered to have degraded microarchitecture by TBS. In women, 38% (*n* = 186) of those considered normal by lumbar spine BMD were considered to have partially degraded or degraded microarchitecture by TBS, and 26% (*n* = 40) of those considered osteopenic by lumbar spine BMD were considered to have degraded microarchitecture by TBS (Fig. 3).

## Discussion

For both men and women, TBS was inversely associated with age. Age was modeled best using a cubic relationship in women and a linear relationship in men. This is similar to the cubic relationship between age and lumbar spine aBMD, which is related to accelerated bone loss during and subsequent to menopause.^2^ This result is not unexpected considering that both TBS and lumbar spine BMD are determined using the same lumbar spine DXA scan. When combined in a best subsets model, age and height were associated with TBS in women, and height and weight were associated with TBS in men. Notably, when unadjusted for age in women, greater TBS was associated with greater height, but lower values for TBS were associated with greater height in the adjusted model. This is likely to be related to the interrelationships between age and height in women. Interestingly, when men were categorised by TBS status, BMD was not different between the normal and partially degraded groups, but was different in the group with degraded microarchitecture. This indicates that TBS has the potential to distinguish variation in fracture risk in those otherwise considered normal by BMD, and therefore capture those in the normal and osteopenic BMD categories who will sustain fractures.

Compared to a study of US non‐Hispanic white women, TBS in the current study was higher in the older age group (*p* < 0.001), but not different in the younger age group (*p* = 0.207).^9^ Another study found TBS of young men and women to be significantly higher than in our study (*p* < 0.001 and *p* = 0.019, respectively).^10^ However, these studies have utilized healthy and volunteer samples which may not be representative of the population. A Japanese study found *T*‐score based cutpoints for TBS in women to be 1.200 (*T*‐score −2.5), and 1.350 (*T*‐score −1.0), which is higher than in the current study, and this may potentially be due to differences in study population or methodology, such as the use of different scanners (Hologic versus Lunar Prodigy).^13^

The current study indicates that approximately 25% of men and women identified by BMD as osteopenic were identified to have degraded microarchitecture by TBS. These individuals classified at high risk by TBS may be important potential targets for fracture prevention, and may contribute to the burden of fracture lying with those in the normal or osteopenia categories when defined by lumbar spine or femoral neck BMD.^3^ The information provided by TBS independent of BMD has been incorporated into Fracture Risk Assessment Tool (FRAX) fracture risk calculations, and has been shown to improve fracture prediction in those close to intervention thresholds.^14^ TBS also has utility in secondary osteoporosis where it may improve fracture risk prediction in patients with type 2 diabetes mellitus and other conditions.^15^ However, it is unclear from the current study whether individuals classified as higher risk by TBS *T*‐scores are in fact those who are likely to sustain fractures.

The data generated from this study will be of particular use in clinical practice, whereby *T*‐scores for TBS can be determined based upon the already captured posterior‐anterior lumbar spine DXA scan, and used in conjunction with lumbar spine and femoral neck BMD measurements to assess fracture risk and make treatment decisions. TBS provides information beyond that given by aBMD, with the potential to capture those in the osteopenic and normal aBMD categories who may fracture, with minimal extra work required by the technician. This makes TBS an attractive tool for use in clinical practice.

This study has many strengths. First, data were taken from a large sample drawn randomly from the population of southeastern Australia, and participation rates were high. Second, the sample covered the full adult age range in both men and women, and these characteristics together provide a strong basis for the development of normative reference ranges that are representative of the population. This is particularly important when it is considered that men are often understudied in osteoporosis research, and further that a representative sample is essential for validity in a clinical setting, where patients will be from the general population. This will be the first population‐based study describing trends of TBS in men. This study also considered potential confounders including height and weight when developing models for TBS. Overall, this study provides a robust reference range for TBS in Australian men and women, particularly where population‐based data for men has not previously been developed.

There were also some limitations. Primarily, the cutpoints developed in this study were based upon equivalent BMD *T*‐score cutpoints, and not from prospective fracture risk in direct relation to TBS. Thus, the ability of these cutpoints to predict fracture is currently unclear. As stated previously, future research validating these reference ranges and cutpoints in a prospective study would be useful. Similarly, lumbar spine BMD was measured as per clinical guidelines from L_2_ to L_4_, whereas TBS was calculated from L_1_ to L_4_, which may affect comparisons. Further, the TBS software used in this study was installed prior to the use of specialised fractal phantoms, which are currently performed at time of installation, and this may have some effect on the TBS values obtained. Finally, due to geographic and other regional variation in bone related measures including TBS, the findings of this study may not be generalizable to other populations.

This study has developed reference ranges for TBS suitable for use in a clinical setting. These reference ranges are distinct for both men and women, with men demonstrating a linear decrease in TBS across the lifespan, whereas the decrease in women is better modeled with a cubic function. *T*‐score–based cutpoints for TBS have also been developed that may be useful for guiding treatment decisions, particularly in individuals close to intervention threshold based upon conventional methods such as BMD. Further research is needed to determine the validity of cutpoints to identify individuals who will fracture.

## Disclosures

DH is co‐owner of the TBS patent and has corresponding ownerships shares and position at Medimaps group. KBA, KLH‐K, MAK, NKH, and JAP have no conflicts of interest to declare.
